# Determinants of metabolic syndrome among patients attending diabetes clinics in two sub-urban hospitals: Bono Region, Ghana

**DOI:** 10.1186/s12872-022-02805-4

**Published:** 2022-08-10

**Authors:** Timothy Agandah Abagre, Delia Akosua Bandoh, Adolphina Addoley Addo-Lartey

**Affiliations:** 1 Department of Epidemiology and Disease Control, School of Public Health, College of Health Sciences, University of Ghana, P. O. Box LG 13, Legon, Accra, Ghana; 2 Ghana Field Epidemiology and Laboratory Training Programme, School of Public Health, College of Health Sciences, University of Ghana, P. O. Box LG 13, Legon, Accra, Ghana

**Keywords:** Type 2 diabetes, Insulin resistance, Metabolic syndrome, Out-patients, Ghana

## Abstract

**Background:**

Over 70% of individuals with type 2 diabetes mellitus (T2DM) may have metabolic syndrome in sub-Saharan Africa. Evidence about the prevalence, clustering, and determinants of metabolic syndrome components is needed to guide the implementation of interventions to prevent cardiovascular diseases in low-income countries.

**Methods:**

A clinic-based cross-sectional study was conducted among 430 out-patients attending two-selected diabetes mellitus clinics in the Bono Region of Ghana. Data was collected in June 2016 among participants aged 30–79 years. The prevalence of metabolic syndrome was assessed using the harmonized definition. Patients were interviewed using semi-structured questionnaires and T2DM status was confirmed by reviewing medical records. The components of MS that were assessed included body mass index, waist circumference, systolic blood pressure, diastolic blood pressure, triglycerides, high-density lipoprotein (HDL)-cholesterol, and blood glucose. Multiple logistic regression models were constructed to evaluate the risk factors of MS.

**Results:**

The mean age of participants was 58.8 ± 11.49 years. The prevalence of MS was 68.6% (95% CI: 64.0–72.8), higher among women (76.3%, 95% CI: 70.6–81.2) than men (58.0%, 95% CI: 35.0–49.4) and in the 50–59-year age group (32.1%). The majority of participants [248 (57.7%)] had either two [124 (28.8%)] or four [124 (28.8%)] components of MS. Excluding fasting blood glucose (78.4%), the predominant components of MS identified in the study were reduced HDL cholesterol (70.2%), high waist circumference (60.9%), and elevated systolic blood pressure (49.8%). The study found that the odds of MS in women are 2.2-fold higher than in men (95% CI: 1.29–3.58, *p* = 0.003). Duration of T2DM (OR 5.2, 95% CI: 2.90–9.31, *p* < 0.001) and overweight status (OR 6.1, 95% CI: 3.70–10.07 *p* < 0.001) were also found to be significant determinants of MS.

**Conclusions:**

Metabolic syndrome was common among patients attending routine diabetes mellitus clinics in sub-urban hospitals in the middle belt of Ghana. Significant factors associated with metabolic syndrome included being female, living with diabetes for more than five years, and being overweight. Nationwide advocacy for routine screening and prevention of the syndrome should be initiated to prevent cardiovascular disease and mortality in this vulnerable population.

## Background

Metabolic syndrome (MS) is a pathologic state characterized by the clustering of metabolic abnormalities [1]. Metabolic syndrome is known by many names including, syndrome X, insulin resistance syndrome, the deadly quartet, the metabolic cardiovascular syndrome, or the atherothrombogenic syndrome [2, 3]. The syndrome (MS) occurs when a person has a combination of any three or more of the following metabolic factors: raised blood glucose or diabetes, high blood pressure, obesity, elevated triglycerides, and low levels of HDL cholesterol.

Global estimates indicate that about a third of the population in every country is affected by MS with predictions of a worldwide increase, primarily in developing countries [1]. Kalk and Joffe reported that 47% of Africans with type 2 diabetes mellitus (T2DM) in South Africa may be affected by MS [4]. Ipadeola and Adeleye have also reported an MS prevalence of over 66% among patients with T2DM in Ibadan, Nigeria [5]. In Ghana, up to 58% of people with T2DM in metropolitan areas may have the syndrome [6]. These estimates are of importance because MS is linked to heart diseases, stroke, and other conditions affecting the blood vessels [7–10]. In adverse conditions, the risk of developing cardiovascular diseases (CVD) and mortality in people with MS is two to three times higher than in those without the syndrome [1, 7, 9].

The concept of MS has existed for many decades albeit with controversies regarding its pathogeneses. Whiles some have associated it with obesity as a result of central adipose tissue [11, 12], others have linked it to insulin resistance [13, 14]. In one review, it was suggested that elevated plasma-free fatty acids in obese subjects induced insulin resistance [15]; an effect known to elicit the development of MS factors [13]. Excess dietary energy has also been implicated in MS pathogenesis [16]. Alemany’s review analyzed the contribution of adipose tissue inflammation to the development of MS and concluded that excess dietary energy (largely fat), elicits insulin resistance, thus creating the problem of excess accumulation of fat in body cells [16].

Despite the diverse schools of thought about the pathogenesis of MS, it appears there is a general leaning towards central obesity as the biggest cause of the syndrome brought in part by the global rise of obesity, consumption of high-energy diets, and sedentary lifestyle [17, 18]. Furthermore, increased age [19, 20], as well as being female have been identified as risk factors in the clustering of MS among individuals with diabetes [21, 22].

While there are pharmacological approaches for the treatment of MS components [23, 24], lifestyle changes are the preferred and sustainable approaches to treating MS [13]. Lifestyle change involves managing all the components in one approach through modifications to personal lifestyle without the use of drugs. The lifestyle approach involves dietary and physical activity modifications, weight management, limiting alcohol, and avoiding smoking [25]. Dietary concepts such as the Mediterranean and Dietary Approaches to Stop Hypertension have also been shown to have protective effects on MS [26–30].

International guidelines for the clinical diagnoses and management of MS have been recommended [31, 32]. At present, there are no standard treatment guidelines for MS in Ghana. This is perhaps the case because of the dearth of data to support the burden of the condition in the country. Further, the lack of a uniform approach for classifying MS has made it problematic when comparing prevalence data across studies. Accordingly, the implications of the information on the burden and associated risk factors of MS depend on the method used. To address the inconsistencies in methods, a group of experts has proposed a harmonized definition for classifying MS [18].

In Ghana, most studies on MS among people with T2DM have been conducted using either the International Diabetes Federation (IDF) or the National Cholesterol Education Program's Adult Treatment Panel III (NCEP: ATP III) definitions [6, 22, 33]. The majority of these studies have been conducted in teaching hospitals in metropolitan areas in the northern or southern regions of Ghana, but less so in sub-urban areas in the middle-belt regions of the country. This study used the new harmonized criterion to investigate the prevalence, distribution of MS components, and associated risk factors among people attending diabetes mellitus clinics in two-selected sub-urban district hospitals in the Bono Region of Ghana.

## Methods and materials

### Study design

The study was an analytical cross-sectional study involving routine diabetes mellitus clinic attendants to determine the prevalence and determinants of MS. Study participants were adult out-patients with known diagnoses of T2DM. Enrolment of participants was done on clinic days when patients attend for care. Data on individual characteristics and exposure to risk factors were collected alongside information about the outcome of interest (MS).

Cochrane’s formula ($ss={z}^{2}*p(1-p)/{d}^{2}$) was used to calculate the sample size needed to determine the prevalence of MS at a 95% confidence interval (z) and 0.05 precision (d). Assuming a *p* of 0.58 (58% prevalence of MS among people with T2DM) [6], the minimum required sample size for this study was calculated to be 379. To cater for nonresponse and/or incomplete questionnaires, an additional 15% was added to give an estimated sample size of 436 participants.

### Study setting

Dormaa Presbyterian and Berekum Holy Family Hospitals are district hospitals located in the Bono Region of Ghana. Both hospitals provide secondary level care services and serve as referral care facilities for cases requiring specialist care from primary health facilities. The two hospitals also provide out- and in-patient services. The average weekly attendance at the Diabetes Mellitus clinic at Dormaa Presbyterian Hospital is 140 patients, while that at the Berekum Holy Family Hospital is 110 patients.

The selection of hospitals for this study was based on the following criteria: (i) That the hospital(s) was a district or municipal hospital not located in the regional capital, Sunyani; (ii) That the hospital(s) had a diabetes clinic with monthly attendance greater than the estimated sample size of 436; (iii) That the hospital(s) had a functioning accredited clinical biochemical laboratory.

### Participants’ recruitment

Participants in Dormaa Presbyterian and Berekum Holy Family Hospitals were recruited on a ratio of 5:4 respectively based on the average weekly clinic attendance. Consequently, 56% (249) and 44% (195) participants were randomly selected for the study at Dormaa and Berekum Hospitals respectively. All T2DM patients attending the hospital's diabetes clinic for care during the study period were considered eligible for participation in the survey. A list of all T2DM clinic attendants was made and a consecutive number from 1 to *n* (number of clinic attendants for a specific day) was assigned to each attendant on the list. Using a random number generator on Microsoft Office excel 2013, a set of random numbers equal to *n*_*1*_ (sub-sample size for the particular clinic, on a specific day) was generated. The generated random numbers were then used to select the participants from the list of attendants. These steps were repeated at each hospital until the sample size was attained.

### Inclusion and exclusion criteria

Participants were included in the study if they were 30 to 79 years of age with clinical diagnoses of T2DM according to WHO criteria [34] regardless of the duration of illness. All study participants were apparently healthy adults including those on treatment for hypertension and diabetes. We included only participants whose diabetes status had been confirmed by a physician and there was information on the patient’s hospital records to confirm the diagnosis. Only those who had consented and were available to participate were included in the research.

Pregnant or lactating mothers were excluded from the study. Also, participants with type 1 diabetes mellitus, history of heart failure, myocardial infarction, hypogonadism, hypothyroidism, acromegaly, and any other chronic diseases were not selected for the study. Patients on prolonged steroid use and those who were on active drug treatment for obesity at the time of the study and critically ill participants, including those with self-reported cardiac problems and amputees, were also excluded from the study.

### Data collection

Data collection was carried out from May to June 2016. The diabetes status of each participant was confirmed with medical records before data collection. Face-to-face interviews were conducted using semi-structured questionnaires to collect participants’ socio-demographic information, including age, sex, educational attainment, marital status, occupation, and duration of T2DM. Information on participants’ lifestyle factors such as engagement in physical activity, alcohol consumption, and dietary habits was obtained through interviews using an adapted semi-quantitative Fenland Food Frequency questionnaire [35]. We also conducted physical measurements to determine participants’ BMI, WC, and blood pressure.

Blood samples of participants were collected from the laboratories of the respective participating hospitals as part of the routine services that diabetes mellitus patients undergo monthly. Each participant’s blood sample was collected between 7:00 and 9:00 AM after 12–14 h of overnight fasting. A sterile single-use syringe and needle set were used to draw blood from each participant’s antecubital fossa. In addition to practicing hand hygiene, laboratory personnel wore fitting non-sterile gloves during blood sample collection. About 5 ml of venous blood was drawn from each study participant and dispensed in serum separator tubes with gel and temporally stored in styrofoam boxes without cold packs but protected from sunlight. The collected blood samples were subsequently centrifuged at 1000 rpm before being analyzed within 24 h after collection. In both Dormaa and Berekum, fasting blood glucose, triglycerides, and HDL-cholesterol levels were assayed enzymatically from blood plasma using Mindray BS-120 automated chemical analyzer.

### Quality control

Data collection was carried out by trained health personnel and entered into pre-defined forms. In addition, blood samples were collected and analyzed by qualified laboratory technicians using standard operating procedures. To ensure the accuracy of data and measurements, all the pieces of equipment, including weighing scales, stadiometers, blood pressure monitors, and blood analyzers were calibrated in accordance with manufacturers’ instructions before data collection. Also, to ensure that blood samples were correctly matched to the results, serum separator tubes were labeled with unique participant identification numbers.

### Variables

The independent variables in this study were the socio-demographic and lifestyle factors, while the outcome variable was MS. Noting that MS results from the clustering of three or more MS components, we determined that the MS components BMI, WC, FBG, BP, TG, and HDL-cholesterol constituted the intermediary variables. All the variables were measured as binary or categorical variables.

### Measurements

A total of 444 respondents were interviewed; however, laboratory, physical and medical information were obtained for 430 participants. Most of the participants 426 (99.1%) were undergoing treatment for only T2DM, while 4(0.9%) of them were on medication for both T2DM and hypertension. To ensure standardization of interviews and measurements, two registered nurses were trained on questionnaire administration, data extraction, and using measurement tools. Participants’ heights and weights were measured using the Seca weighing scale and stadiometer respectively. A simple tape measure calibrated in meters to the nearest centimeter was used to measure waist circumference on the bare skin at the end of normal gentle respiration. Participants were required to wear light clothing and without shoes during physical measurements. Measurement of waist circumference was taken at the narrowest indentation midway between the lowest rib and the iliac- crest.

The average of two blood pressure readings was taken after five minutes apart using Omron electronic blood pressure monitor. Measurements were taken in the sitting position after participants have had at least 15 min of rest. Participants’ blood samples were also collected and tested for glucose, triglycerides, and HDL-cholesterol level after which the results were documented in the questionnaires.

### Classification of metabolic syndrome

Metabolic syndrome (MS) was defined according to the new harmonized definition [18]. Based on this criterion, a person has MS if s/he has three or more of the following: elevated waist circumference of > 94 cm for men or > 88 cm for women; elevated triglycerides (or treatment for elevated triglycerides) ≥ 150 mg/dL (1.7 mmol/L); reduced HDL-cholesterol (or treatment for reduced HDL) < 40 mg/dL (1.0 mmol/L) in males and < 50 mg/dL (1.3 mmol/L) in females; elevated blood pressure (or history of hypertension)—systolic ≥ 130 and/or diastolic ≥ 85 mm Hg; and elevated fasting glucose ≥ 100 mg/dL (or treatment of elevated glucose).

#### Statistical analysis

Data analysis was done using Stata version 13.1. This study had a response rate of 96.8%. The mean and standard deviation was used to describe continuous variables such as age, waist circumference, blood glucose, blood pressure, high-density lipoprotein cholesterol, and triglycerides. Discrete variables such as the sex- and age-specific prevalence of MS and its components were presented as proportions and 95% confidence intervals (CI). We performed the *chi-square* test on categorical variables to test the association between potential risk factors and MS. For continuous variables, the *Students t-test or Wilcoxon rank-sum* test was used in assessing the difference in mean measurements between men and women and by the MS status. Two logistic regression models were performed to test the strength of association between risk factors and MS. Despite the fact that hypertension is one of the confounding factors for the development of MS, we did not stratify our analyses based on the presence or absence of diabetes and hypertension or exclude those with hypertension from the analyses. This was due to the fact that the study only included four individuals from the Dormaa Presbyterian Hospital who had just received a diagnosis of hypertension and were being treated for both it and T2DM. Because they had only obtained a diagnosis during the month prior to the data collection and because their numbers were deemed insufficient to significantly affect the study's conclusions, they were recruited into the study and included in all analyses. Simple logistic regressions were carried out to test the crude relationships between exposures and MS. Multiple logistic regression analyses were then used to evaluate the adjusted relationship between risk factors and MS. Statistical significance was assumed if *p*-values were < 0.05.

#### Ethical considerations

Ethical clearance for the study was obtained from the Ghana Health Service Ethical Review Committee (GHS-ERC: 08/12/15). Permission was also sought from the hospital authorities at Dormaa Presbyterian Hospital and Berekum Holy Family Hospital respectively. Informed consent was delivered verbally and respondents were asked to sign or thumb-print the informed consent if they agreed to be enrolled in our study. All methods were performed in agreement with the relevant guidelines and regulations.

## Results

### Demographic, anthropometric, and biochemical characteristics of participants

This analysis involved 430 people with T2DM of whom 249 (57.9%) were women (Table 1). The majority of participants 426 (99.1%) had only T2DM as the main diagnosis, while 4(0.9%) had both T2DM and hypertension. There were more married (66.0%) participants than divorced, separated, or never married (34.0%) participants. Farming was the main occupation for 258 (60.0%) of participants. Those who were formally employed were 27 (6.3%), while 66 (15.4%) were unemployed or retired. The rest of the participants were traders, carpenters, or construction workers. Most participants [383 (89.1%)] in this study had less than senior high school education. The age of respondents ranged from 30 to 79 years with the majority [138 (32.1%)] of participants in the 50–59 years age category, while the minority [24 (5.6%)] of them were in the 30–29 years age group.

**Table 1:** Characteristics of study participants and prevalence of MS

	Participants (*N* = 430)		Metabolic syndrome (*N* = 295)			
Parameter	*N*	%	Prevalence	%	95% C.I	*p-*value
Sex						< 0.000
Men	181	42.1	105	42.0	35.0–49.4	
Women	249	57.9	190	76.3	70.6–81.2	
Marital status						0.009
Married	284	66.0	183	64.4	58.7–69.8	
Divorce/Not married	146	34.0	112	76.7	69.1–82.9	
Age categories						0.697
30–39	24	5.6	16	66.7	44.5–83.3	
40–49	60	14.0	46	76.7	64.0–85.9	
50–59	138	32.1	93	67.4	59.0–74.8	
60–69	113	26.3	75	66.7	57.0–74.6	
70–79	95	22.1	65	68.4	58.2–77.1	
Occupation						0.212
Farmers	258	60	173	67.1	61.0–72.6	
Traders	64	14.9	52	81.3	69.5–89.2	
Office-related	27	6.3	17	63.0	42.5–79.7	
Unemployed	66	15.3	43	65.2	52.6–75.9	
Other	15	3.5	10	66.7	37.2–87.1	
BMI						
Normal/underweight	207	48.1	106	51.2	44.4–55.6	< 0.001
Overweight/Obesity	223	51.9	189	84.8	79.4–88.9	
Educational status						0.679
Less than SHS education	383	89.1	264	68.9	64.1–73.4	
SHS or higher	47	10.9	31	66.0	50.9–78.4	
Family history of T2DM						
Yes	306	71.2	214	69.9	64.5–74.8	0.351
No	124	28.8	81	65.3	56.2–73.3	
Duration of T2DM						< 0.001
Less than 5 years	267	62.1	156	58.4	52.4–64.2	
5 years and above	163	37.9	139	85.3	78.9–90.0	
Physical activity						0.153
Three or more times/week	98	22.8	73	74.5	64.8–82.3	
Less than three times/week	332	77.2	222	66.9	61.6–71.7	
Fruits						0.368
Daily	22	5.1	14	63.6	42.2–80.7	
Not daily	408	94.9	281	68.9	64.2–73.2	
Vegetables (excludes tomatoes)						0.946
Daily	82	19.1	56	68.3	57.3–77.6	
Not daily	348	80.9	239	68.7	63.6–73.4	
Oil-cooked foods						
Daily	64	14.9	48	75.0	63.0–84.1	0.254
Not daily	366	85.1	247	67.5	62.5–72.1	
Alcohol consumption						
At least occasionally	21	4.9	13	61.9	40.2–78.7	0.498
Don’t drink	409	96.1	282	68.5	64.2–73.3	
Total	430	100.0	295	68.6	64.2–72.8	

SHS Senior High School

On lifestyle-related factors, 21 (4.8%) of participants reported that they consumed any type of alcohol once or less than once weekly, while the rest 409 (96.1%) reported that they did not consume any type of alcohol. Participants who engaged in moderate to vigorous physical activity at least 3 times per week were 98 (22.8%). The participants who reported that they consumed fruits daily were 22 (5.1%), while 82 (19.1%) reported consuming vegetables (except tomatoes) daily. Participants who reported consuming oil-cooked foods such as fried foods and stews four to six times a week were 64 (14.8%).

Table 2 shows the mean anthropometric and biochemical measurement of participants by sex and metabolic syndrome status. The mean age of participants was 58.84 ± 11.49 years. Men had a higher mean age (60.39 ± 11.46 years) than women (57.71 ± 11.40 years), *p* = 0.015 (Table 2). All participants had previous diagnoses of T2DM with an average disease duration of 5.30 ± 3.84 years. The mean BMI of participants was 25.76 ± 4.74 kg/m^2^. The six MS components measured in this study were WC, SBP, DBP, triglycerides, HDL, and blood glucose. Except for triglyceride levels, there were no sex differences in the mean measurements of the remaining MS component. Men had higher mean triglyceride level (127.12 ± 50.28 mg/dl) than women (143.08 ± 57.24 mg/dl), *p* = 0.004. Between participants with MS and those without MS, the difference in mean measurements for each component of MS was significant for all the components (*p* < 0.001) except FBG (*p* = 0.400).

**Table 2:** Mean physical and biochemical measurement of participants by sex and metabolic syndrome

	Total	Male	Female	*P*-value	MS Present	MS Absent	*P*-value
Parameter	Mean ± SD	Mean ± SD	Mean ± SD				
Age (years)	58.84 ± 11.49	60.39 ± 11.46	57.71 ± 11.40	0.015	58.71 ± 11.50	59.14 ± 11.50	0.716
Duration of T2DM (years)	5.30 ± 3.84	5.12 ± 3.42	5.41 ± 4.11	0.973	5.79 ± 3.92	4.22 ± 3.43	< 0.001
BMI (kg/m^2^)	25.76 ± 4.74	25.60 ± 4.17	25.87 ± 5.11	0.573	26.99 ± 4.69	23.06 ± 3.60	< 0.001
WC (cm)	91.37 ± 11.34	91.86 ± 10.23	91.02 ± 12.11	0.447	94.83 ± 10.50	83.82 ± 9.31	< 0.001
SBP (mm/Hg)	134.06 ± 20.35	134.54 ± 21.02	133.72 ± 19.87	0.674	138.94 ± 20.42	123.40 ± 15.61	< 0.001
DBP (mm/Hg)	82.48 ± 10.72	82.27 ± 10.51	82.63 ± 10.89	0.729	84.85 ± 10.75	77.30 ± 8.68	< 0.001
TG (mg/dL)	136.36 ± 54.92	127.12 ± 50.28	143.08 ± 57.24	0.004	148.52 ± 58.36	107.60 ± 31.17	< 0.001
HDL (mg/dL)	35.87 ± 15.41	34.31 ± 15.13	37.01 ± 15.54	0.061	33.20 ± 1438	41.71 ± 16.00	< 0.001
FBG (mg/dL)	137.97 ± 54.22	135.69 ± 54.43	139.62 ± 54.11	0.385	139.97 ± 52.31	134.71 ± 58.23	0.400

*WC* Waist circumference, *SBP* Systolic Blood Pressure, *DBP* Diastolic Blood Pressure, *TG* Triglycerides, *HDL* High-density lipoprotein, *FBG* Fasting Blood Glucose

### Prevalence of metabolic syndrome

Metabolic syndrome was present in 295 (68.6%) of participants (95% CI: 64.0 -72.8) (Table 3). The prevalence of the syndrome was higher among women [190 (76.3%)] than men [105 (58.0%)], *p* < 0.001 (Table 1). MS was also more prevalent among unmarried (divorced, separated, or never married) respondents [112 (76.7%)] than married participants [183 (64.4%)], *p* = 0.009. The prevalence of MS increased from 5.6% in the 30–39-year age group to 14.0% in the 40–49 years age group and peaked in the 50–59-year age group (32.1%) before declining to 22.1% in the 70–79-year age group. The relationship between respondent age group, occupation, or education, and MS was not significant (*p* > 0.05) (Table 1). The mean age of participants was not different between participants with MS and those without MS (*p* = 0.716).

**Table 3:** Clustering of metabolic syndrome components among participants

Type/No. of MS components	Male (No.)	% (95% CI)	Female (No.)	% (95% CI)	Total (No.)	% (95% CI)
MS component						
High TG	44	24.3 (18.6–31.2)	95	38.2 (32.3–44.4)	139	32.3 (28.1–36.9)
High DBP	65	35.9 (29.2–43.2)	91	36.5 (30.8–42.8)	156	36.3 (31.9–41.0)
High SBP	95	52.5 (45.1–59.7)	119	47.8 (41.6–54.0)	214	49.8 (45.0–54.5)
Elevated WC	75	41.1 (34.4–48.8)	187	75.1 (69.3–80.1)	262	60.9 (56.2–65.4)
Elevated FBG	138	76.2 (69.4–81.9)	199	79.9 (74.4–84.5)	337	78.4 (74.2–82.0)
Low HDL	119	65.4 (58.5–72.4)	183	73.5 (67.6–78.6)	302	70.2 (65.7–74.4)
Obese/overweight	92	50.8 (43.5–58.1)	137	55.0 (48.8–61.1)	229	53.3 (48.5–57.9)
No. of components present						
1	5	2.8 (1.1–6.5)	11	4.4 (2.4–7.8)	16	3.7 (2.2–6.0)
2	74	40.9 (33.9–48.3)	50	20.1 (15.5–25.6)	124	28.8 (24.7–33.3)
3	53	29.3 (23.1–36.4)	63	25.3 (20.3–31.1)	116	26.9 (23.0–31.4)
4	38	21.0 (15.6–27.6)	86	34.5 (28.9–40.7)	124	28.8 (24.7–33.3)
5	11	6.1 (3.4–10.7)	39	15.7 (11.6–20.8)	50	11.6 (8.9–15.0)
≥ 3 (MS)	105	42.0 (35.0–49.4)	190	76.3 (70.6–81.2)	295	68.6 (64–0-0.72.8)

Participants who had lived with T2DM for five or more years had a higher prevalence of MS [139 (85.3%), 95% CI: 78.9–90.0] than their counterparts who had lived with the condition for less than five years [156 (58.4%), 95% CI: 52.4–64.2], *p* < 0.001. The difference in the mean duration of T2DM between participants with MS and those without MS was present in significant, *p* < 0.001 (Table 3). Among participants with a family history of T2DM, MS was observed in 214 individuals (69.9% CI: 64.5–74.8), while for those without a family history of diabetes, MS was common in 81 persons (65.3%, 95% CI: 56.2–73.3), *p* = 0.351.

### Clustering of metabolic syndrome components

The majority of participants [248 (57.7%)] had either two [124 (28.8%)] or four [124 (28.8%)] components of MS (Table 3). A total of 16 participants (3.7%) had one MS component, while 116 (26.9%) had three MS components. Those who had the maximum of five MS components were [50 (11.6%)]. Besides FBG, the top three predominant MS components were reduced HDL-cholesterol [302 (70.2%)], elevated waist circumference [262 (60.9%)] and high SBP [214 (49.8%)]. Reduced HDL-cholesterol (46.3%) high SBP (22.1%) and elevated WC (17.4%) were the most frequently occurring MS components among men. In women, elevated WC (43.5%), reduced HDL-cholesterol (42.6%) and high SBP (27.7%) were the three predominant components.

### Determinants of metabolic syndrome

In the crude logistic regression analyses, factors such as sex, marital status, duration of T2DM, overweight/obesity, and being a trader were found to be associated with MS to varying extents (Table 4). The crude analysis indicated that the odds of MS were 2.3 times higher among women than men (95% CI: 1.53- 3.53), *p* < 0.001. Unmarried participants had 1.8 times the odds of MS compared with married participants (95% CI: 1.15–2.86), *p* < 0.001. Participants who had lived with T2DM for five or more years were 4.1 times as likely to have MS compared with participants who had lived with MS for less than five years (95% CI 2.51–6.77, *p* < 0.001). Overweight/obese participants were also more likely to have MS than normal or underweight participants [crude OR: 5.5 (95% CI: 1.15–2.86, *p* < 0.001)]. Compared with farmers, respondents who were traders had 2.1 times the odds of having MS (95% CI: 1.08–4.20, *p* = 0.029).

**Table 4:** Factors associated with MS among people with T2DM

	Simple logistic regression			Multiple logistic regression		
Parameter	Crude OR	95% CI	*p*-value	Adjusted OR	95% CI	*p*-value
*Sex*						
Men	Reference			Reference		
Women	2.33	1.53–3.53	< 0.001	2.15	1.29–3.58	0.003
*Duration of T2DM*						
Less than 5 years	Reference			Reference		
More than 5 Year	4.12	2.51–6.77	< 0.001	5.20	2.90–9.31	< 0.001
*Obesity status*						
Obesity statusNot overweight/obese	Reference			Reference		
Obesity statusOverweight/obese	5.49	3.49–8.64	< 0.001	6.10	3.70–10.07	< 0.001
*Age (years)*						
Age (years)30–39	Reference					
40–49	1.64	0.58–4.64	0.349			
50–59	1.03	0.41–2.59	0.944			
60–69	0.99	0.39–2.51	0.978			
70–79	1.08	0.42–2.81	0.869			
*Marital status*						
Married	Reference					
Unmarried	1.82	1.15–2.86	0.010			
*Education*						
Less than secondary	Reference					
Secondary and higher	0.87	0.46–1.66	0.679			
*Occupation*						
Farmers	Reference					
Traders	2.13	1.08–4.20	0.029			
Office-related	0.84	0.37–1.90	0.668			
Unemployed/pensioner	0.92	0.51–1.62	0.770			
Other	0.98	0.33–2.97	0.975			
*Family history of T2DM*						
No	Reference					
Yes	1.24	0.79–1.92	0.351			
*Physical activity*						
3 or more times/week	Reference					
Less than 3 times per week	1.45	0.87–2.41	0.154			
*Fruit consumption*						
Daily	Reference					
Not daily	1.26	0.52–3.09	0.607			
*Vegetable consumption*						
Daily	Reference					
Not daily	1.02	0 .61–1.71	0.946			
*Eating oil-cooked food*						
Not daily	Reference					
Daily	1.44	0.79–2.65	0.234			

In the adjusted analyses, sex, duration of T2DM, and Obesity status are each adjusted for all the other covariates in the simple logistic regression analyses

In the multiple logistic regression model, all the exposures in the simple logistic regression model were controlled for each other as potential confounders. The adjusted results showed that sex, duration of T2DM, and overweight/obesity were associated with the odds of developing MS (Table 4). The odds of developing MS among women were 2.15 times that of men (95% CI: 1.29–3.58, *p* < 0.003). Compared with normal or underweight participants, overweight/obese participants were more likely to have MS [adjusted OR: 6.1 (95% CI: 3.70–10.07, *p* < 0.001)]. The adjusted odds of MS in respondents who had had T2DM for 5 or more years was 5.2 times compared to those with a disease duration of lesser than 5 years (95% CI: 2.90–9.31, *p* < 0.001).

## Discussion

This study assessed the prevalence and risk factors of MS among people attending diabetes mellitus clinics using the harmonized criterion. MS was defined as the presence of at least any three abnormal MS components. The results indicate that MS is a common occurrence (68.6%) among people with T2DM who attend routine clinics in suburban hospitals in the middle-belt region of Ghana. The prevalence of MS was much higher in this study than in the earlier studies conducted in other parts of Ghana. For instance, using the NCEP: APT III definition, Titty (2010) conducted a prospected study among 240 patients recently diagnosed with diabetes mellitus in the Tamale Teaching Hospital and found that MS was present in 43.3% of the cohort [33]. In another study at the Komfo Anokye Teaching Hospital in Kumasi, Nisiah et al*.* (2015) conducted a cross-sectional study using the NCEP: ATP III definition of MS and reported an MS prevalence of 58.0% among 150 participants with T2DM [33]. Although the harmonized and NCEP: ATP III definitions of MS are similar, there is a key difference in terms of the cut-off points for elevated fasting blood glucose and high blood pressure. These cut-offs (being slightly lower in the Harmonized definition), might explain the higher prevalence of MS recorded in our study. In contrast, Mogre et al*.* (2014) recruited 200 participants with T2DM for a cross-sectional study at the Tamale Teaching Hospital and found that the prevalence of MS was 24.0% among participants using the IDF definition [22]. The prevalence of MS in the Mogre et al. study might have been underestimated given that the investigators excluded triglycerides and HDL-cholesterol components in their diagnosis of MS.

The prevalence of MS in this study was also found to differ from those of studies conducted in other African countries. In one cross-sectional study among 254 patients with T2DM in a teaching hospital in Nigeria, the prevalence of MS defined by the WHO criterion was reported in 59.0% of participants [36]. In South Africa, using the IDF definition, MS prevalence of 46.5% and 74.1% were reported among black and white South African patients with T2DM respectively [4]. Ipadeola & Adeleye (2015) also conducted a study at a University Hospital in Ibadan, Nigeria, and found that MS prevalence was 66.0% among 340 participants with T2DM when classified by the IDF definition [5]. Among people with T2DM in Cameroon, the prevalence of MS was 71.7% as defined by the IDF criterion and 60.4% using the NCEP-ATP III definition in the same study [37].

Beyond the study designs, sample sizes, and ethnic or geographical differences, the spatial variability of MS prevalence across studies conducted in Ghana and the African continent might have been influenced by differences in approaches used to measure MS. Depending on the type of criterion used to diagnose MS, the prevalence of the syndrome might differ between studies [38]. Reports from several studies suggest that the harmonized criterion is a more sensitive indicator of MS than the IDF, WHO, or NCEP: APT III definitions of MS [39–41]. This might have explained why the prevalence of MS was higher in this present study than in the earlier studies conducted in Ghana [6, 22, 33].

Few studies have assessed the prevalence of MS among people with T2DM using the harmonized definition. In Nigeria, Ogbera et al. (2010) recruited patients with T2DM from an urban hospital and reported an 86.0% prevalence of MS among participants using the harmonized definition [42]. In one Nepalese study involving 1061 patients with T2DM, the prevalence of MS was reported in 80.3% of participants [39]. A similar study conducted among participants with T2DM in India reported an MS prevalence of 71.9% [9]. The disagreements in the prevalence of MS between these studies and our findings could be attributed to ethnic and/or geographic differences.

Analysis of the risk factors of MS in this present study revealed sex differences in the prevalence of MS. It was observed that women were more likely to be diagnosed with MS than men. This finding is in agreement with the observations from several studies conducted in Ghana [6, 22, 33]; other parts of Africa [4, 5]; and elsewhere [9, 43]. However, one study conducted in India reported higher odds of MS in men compared with women [44]. The higher odds of MS among women than in men observed in this present study could be attributed to a higher prevalence of obesity in women than in men. This is supported by evidence from several studies that have linked obesity to MS [45–47]. Other studies have linked differential clustering of metabolic factors in women and men to hormonal changes in adulthood [48], and the influence of genetic backgrounds, diet, level of physical activity, and under or over-nutrition [38].

Another important risk factor of MS that was identified in this study was the duration of T2DM. The findings of this study reveal that living with T2DM for more than five years had a positive independent association with MS (*p* < 0.001). Our observation is in agreement with the Mogre et al. (2014) study conducted among people with T2DM in a teaching hospital in northern Ghana [22]. Similarly, a study among 159 diabetes patients in Ethiopia found that having diabetes for over five years was significantly associated with MS [49]. The authors suggested that lack of awareness and inadequate health care for people with T2DM was likely to be implicated in their findings. In contrast, a study in Japan [50] and Hong Kong [51] found that living longer with T2DM correlated positively with the prevalence of MS. Shimajiri et al*.* (2008) attributed the inverse relationship between MS prevalence and duration of T2DM to improved BMI brought about by enhanced medical care and improved metabolic control [50]. This is understandable given that stronger health care systems in such developed countries could lead to improved health outcomes for people with T2DM. In this present study, the negative association between the duration of T2DM illness and MS may be due to poor metabolic and glycaemic control as a result of inadequate medical care.

This present study also revealed differential patterns of MS between overweight/obese participants and normal-weight participants. Participants who were overweight/obese were more likely to have MS than their counterparts who were not overweight/obese. A similar study conducted among participants with T2DM in Nigeria using the harmonized definition found MS to be significantly associated with obesity [42]. Population-based studies in the United States of America using the NCEP/ATP III definition have also reported obesity to be independently associated with MS [47, 52]. These observations are not unexpected given that obesity is a known risk factor for MS [45, 46].

Contrary to existing evidence, this present study did not associate MS with increased age [22, 48, 52, 53], or educational attainment. Similar studies conducted among T2DM patients in Nigeria using the IDF criterion [5], and India using the harmonized criterion [9, 44] also did not associate MS with increased age. In contrast, several cross-sectional studies among people with T2DM [22, 53] and in the general population [48, 52] using various definitions of MS have reported age differences in the way MS is expressed. In terms of the relationship between MS and educational attainment the results of this study are comparable with the Bhatti et al. (2015) study among T2DM patients in India [9], but at variance with the Nsiah et al. (2015) study among diabetes patients in southern Ghana [6]. While the mechanism explaining the lack of association between MS and increased age or educational attainment in our study is unclear; it could have been influenced by similarities in physical activity and under-or over-nutrition levels between younger and older participants.

The three most common constituents of MS among participants in the study were reduced HDL-cholesterol levels, elevated FBG (hyperglycemia), and high SBP. Given that all the study participants were patients living with T2DM, we excluded elevated blood glucose levels in the assessment because it is the main indicator of diabetes. If raised FBG had not been excluded, it would have been the predominant component of MS among study participants. After excluding elevated FBG from the assessment, reduced HDL-cholesterol levels emerged as the most prevalent component of MS in the present study. This observation was consistent with the results from one study in Libya that used the WHO and IDF definitions to define MS [54]. In contrast, two earlier studies among people with T2DM in Ghana used the IDF [22] and the NCEP/ATP III [6] definitions of MS and reported WC and high blood pressure as the predominant components of MS respectively. In Nigeria, Ogbera et al*.* (2010), used the harmonized criterion and found WC to be the most commonest component of MS among study participants with T2DM [42]. Nonetheless, reduced HDL-cholesterol was among the three principal components of MS in these earlier studies [6, 22, 42]. The high prevalence of reduced HDL cholesterol among participants in this study could be a consequence of uncontrolled hyperglycemia among participants in this study. Eckel et al*.* (2005) have suggested that people with elevated blood glucose readings may also present with reduced HDL cholesterol [13].

Given that obesity has strong links with MS [45–47], it was not unexpected to find increased WC as one of the three most common components of MS in this study. Similar studies among people with T2DM in Ghana [22] using the IDF classification; Nigeria [42] using the new harmonized definition; and Seychelles [55] using the IDF, APT, and WHO definitions, have also reported WC among the three predominant components of MS. In contrast, among non-obese individuals with MS in India, Dhanaraj et al. (2008) found elevated serum triglycerides in men and low serum HDL-cholesterol in women, but not WC, to be the strongest determinant of MS using a modified APT III classification of MS [56].

In agreement with the results of this study, several studies have also reported raised SBP among the three commonest components of MS [6, 9, 57]. Using the NCEP/ATP III criterion, Nsiah et al. (2015) conducted a cross-sectional study among people with T2DM in a teaching hospital in southern Ghana and reported SBP as the commonest component of MS [6]. In Nigeria, a hospital-based study assessing MS among subjects with T2DM also reported SBP as the predominant component of MS among participants [57]. Similarly, a population-based study assessing MS among urban diabetic patients in northern India found SBP to be the commonest component of MS among participants in the study [9]. The evidence appears to suggest that when hyperglycemia is excluded, reduced HDL-cholesterol, elevated WC, and raised blood pressure emerged as the common MS components that coexist in people with T2DM [11, 58].

This study has some limitations and strengths to note. Owing to the cross-sectional design, this study was unable to establish temporal associations between the explanatory factors and the occurrence of MS. Additionally, given that all participants were selected from a hospital setting, the findings of this present study might not be representative of all persons with T2DM. Nonetheless, we believe our findings are reflective of MS prevalence and its associated risk factors among patients with T2DM and akin populations because of the relatively large sample size of the participants. Also, using the new harmonized definition for metabolic syndrome aligns our research with current international guidelines for evaluating MS.

## Conclusions

The findings of this study support the evidence from earlier studies that suggest that MS is widespread among routine clinic attendants with T2DM in Ghana. Consistent with the other studies conducted in Ghana and similar settings, this study found MS to be more common in women than in men. In addition, the duration of T2DM and overweight/obesity status were identified as important risk factors of MS in our population. The three most predominant components of MS identified in this study were low HDL cholesterol, high waist circumference, and elevated SBP. Tackling the MS epidemic requires targeted interventions to address these specific components of MS among people with T2DM.

### Implications

The burden of MS in the present study reflects a need for the healthcare system (Ghana Health Service) to develop specific protocols and systems for the routine diagnosis and management of MS for people with T2DM in hospitals across Ghana. Priority should be placed on testing for the most predominant components identified in this and other relevant studies. There is also the need for hospitals to set up diabetes management teams to ensure close monitoring and treatment of emerging metabolic factors among people with T2DM. Finally, to effectively prevent, control, and manage MS in Ghana, the Ghana Health Service should establish a surveillance system for MS in all hospitals across the country.

## Data Availability

The dataset for this present study is available from the corresponding author (aaddo-lartey@ug.edu.gh) upon reasonable request.

## Abbreviations

ATP: Adult treatment panel
BMI: Body mass index
CVD: Cardiovascular disease
DBP: Diastolic blood pressure
DM: Diabetes mellitus
FBG: Fasting blood glucose
HDL: High-density
IGT: Impaired glucose tolerance
IDF: International diabetes federation
MS: Metabolic syndrome
NCEP: National cholesterol education program
SBP: Systolic blood pressure
T2DM: Type 2 diabetes mellitus
TG: Triglyceride
WC: Waist circumference
